# Association of polymorphism of IL‐17A, IL‐17F, and IL‐6 with *Toxoplasma gondii* infection susceptibility in **HIV/AIDS** patients in Shiraz, southern Iran

**DOI:** 10.1002/iid3.1117

**Published:** 2024-01-10

**Authors:** Maryam Nejabat, Mohammadreza Heydari, Mohammad Motamedifar, Zohre Foroozanfar, Saeid Amirizadeh Fard, Ava Hashempour, Nazani Nazari, Esmaeil Rezaei, Zahra Heydari

**Affiliations:** ^1^ HIV/AIDS Research Center, Institute of Health Shiraz University of Medical Sciences Shiraz Iran; ^2^ Department of Bacteriology and Virology, Shiraz Medical School Shiraz University of Medical Sciences Shiraz Fars Iran; ^3^ Virology Section, Diagnostic Laboratory Sciences and Technology Research Center School of Paramedical Sciences Shiraz Iran; ^4^ Department of Immunology, Shiraz Medical School Shiraz University of Medical Sciences Shiraz Iran; ^5^ Department of Biochemistry, Faculty of Biological Sciences Tarbiat Modares University Tehran Iran; ^6^ Department of Cell and Molecular Biology, Faculty of Life Sciences and Biotechnology Shahid Behesti University Tehran Iran

**Keywords:** HIV/AIDS patients, IL‐17A, IL‐17F, IL‐6, Toxoplasma gondii infection

## Abstract

**Introduction:**

*Toxoplasma gondii* infection is considered as one of the most important opportunistic infections and cause of death in HIV patients.

**Methods:**

In this cross‐sectional study, 334 HIV positive patients were included. The molecular test was performed by the restriction fragment length polymorphism–polymerase chain reaction method. Allelic frequency, haplotype analyses, and linkage disequilibrium were calculated. The odds ratio was calculated. The linear regression model was used to analysis of interleukin (IL)‐17A, IL‐17F, and IL‐6 single‐nucleotide polymorphism genotypes in HIV patients with and without toxoplasmosis.

**Results:**

In total, 95 tested'patients (28.4%) were positive for toxoplasmosis. The risk of *toxoplasma* infection in the current study did not correlate with IL‐17 and IL‐6 polymorphism and the risk of contracting *toxoplasma* was also not significantly correlated in this study. There was no association between the frequency of alleles and the risk of toxoplasma infection in IL‐17 haplotype analysis.

**Conclusion:**

The findings of this study revealed that there were significant differences in the serum levels of IL‐6 and IL‐17A, but not IL‐17F, between the case and control groups in various genetic models. However, these polymorphisms did not show a significant relationship with toxoplasma infection in HIV‐positive patients. This study represents the first investigation in Iran to explore the role of IL‐6 and IL‐17 polymorphisms in toxoplasma infection among HIV‐positive patients.

## 1 INTRODUCTION

*Toxoplasma gondii* (*T. gondii*), an obligate intracellular protozoan with a predilection for the placenta, central nervous system, and human eye, distributed in human and animal societies worldwide; therefore, it is considered as one of the most zoonotic parasites of human.^1^ This organism can be transmitted in three ways: consumption of contaminated raw or undercooked meat products, consumption of water and food contaminated with infectious oocysts, and congenital transmission.^2^, ^3^

The incidence of *T. gondii* infection varies with geographic areas, weather conditions, and contact with cats. In addition, some risk factors such as occupation, gender, place of residence, and level of education are indirectly effective in contagion and cause a change in the infection pattern.^4^

Although infection with *T. gondii* appears to be asymptomatic or present as a self‐limiting disease in healthy individuals, but it has the potential to cause severe disease in immunocompromised patients and be fatal in this population.^5^ *T. gondii* infection is considered as one of the most important opportunistic infections and the leading cause of death in patients with HIV. In this group of people, reactivation of tissue cysts and recurrence have occurred due to the deficient body's immune response, and therefore the prevalence of toxoplasmosis infection in these people will be higher than normal population.^6^

The most common manifestation of *T. gondii* infection in immunocompromised patients is toxoplasmic encephalitis, which causes fever, headache, convulsions, and decreased consciousness, as well as memory loss and even death. Also, these people are more likely to have severe ocular toxoplasmosis.^7^, ^8^

The prevalence of *T. gondii* infection in HIV patients varies worldwide.^9^ The overall reported rate of seroprevalence around the glob is 35.8%. Still, this statistic varies by geographic region (60.7% in the Middle East and North Africa, 49.1% in Latin America, 44.9% in Sub‐Saharan Africa, 30.1% in Western Europe and Central and North America, and 25.1% are reported in Asia).^5^

According to the results of a meta‐analysis, the overall prevalence of toxoplasma infection among people with immune system defects in Iran is estimated about 50%, which the most vulnerable groups being people with organ transplants 55%, people with HIV 50% and people with cancer 45% percent.^3^, ^10^

Cellular immunity is a key component of the host immune response against *Toxoplasma*. Macrophages, T lymphocytes, natural killer (NK) cells, and cytokines are the main elements involved in the immune response. The strength of the immune system's responses to infection is tightly regulated by the activities of chemokines, cytokines, and their receptors.^11^

Following infection with *T. gondii*, proinflammatory cytokines, including interleukin (IL)‐1, IL‐6, IL‐12, and tumor necrosis factor, as well as IL‐17, have been reported to be involved in the development of immune responses. Functional polymorphisms of cytokines may play an important role in the regulation of inflammatory activities and resistance to infectious diseases.^12^, ^13^

The most commonly studied polymorphism in IL‐6 is the single‐nucleotide polymorphism (SNP) 6‐174G/C. Studies have shown that IL‐6 (G/C) SNPs may play an essential role in the exacerbation and progression of toxoplasmosis infection. IL‐6 is a multifunctional proinflammatory cytokine belonging to the IL‐1 cytokine family. This IL mediates several critical physiological functions, including the control of the acute phase response during inflammation.^14^

Previous studies have identified an important role for IL‐6 in resistance to *T. gondii*, the genetic modifications from SNPs of IL‐6 genes, may have been associated with susceptibility to *T. gondii* infection,^15^ although the mechanism by which IL‐6 enhances resistance to this pathogen is unclear.^16^ It has also been shown that IL‐6 gene polymorphisms cause a decrease in the plasma concentration of this IL and increase in the prevalence of toxoplasmic retinochoroiditis.^17^

IL‐17 is a cytokine from the proinflammatory cytokine family secreted by helper T cells and causes the production of proinflammatory cytokines and the recruitment of neutrophils and monocytes. IL‐17 plays an important role in the development and progression of inflammatory and autoimmune diseases.^18^ However, studies have shown that this IL plays a protective role against *Toxoplasma*. The gene regions IL‐17A rs 2275913 and IL‐17F rs 763780 are two important polymorphisms of this IL. IL‐17A is considered as an important player in host protection against extracellular and some intracellular pathogens.^19^ NK cells, CD4+, and CD8 cells are the main sources of IL‐17 production during toxoplasmosis. This inflammatory cytokine induces innate immunity by recruiting neutrophils that render the host resistant to *T. gondii* infection.^11^

In IL‐17 receptor‐deficient mice infected with *T. gondii*, after increasing the parasite, burden is accompanied by a decrease in neutrophil counts and CXCL8 expression, which reduces animal survival.^12^ The SNP of IL‐17A has been found to have different associations with recurrent abortion women infected with toxoplasmosis. In the case of recurrent abortion women with toxoplasmosis, the genotype AA and allele A of IL‐17A have been identified as protective factors. On the other hand, in recurrent abortion women (without toxoplasmosis), the allele G of IL‐17A has been identified as a risk factor,^20^ but we not found any study in non‐ pregnant population.

In addition, toxoplasmainfected mice with IL‐17 deficiency show higher mortality compared to mice with normal levels of this IL. This is due to the reduction in neutrophil absorption during *Toxoplasma* infection and the increase in parasite load.^21^

Toxoplasmosis and HIV infections are major public health problems worldwide and pose a challenge to Iran's health problems.^22^ Due to the increase in the prevalence of patients with immune deficiency, there is a need to gather information on the prevalence of *T. gondii* and the monitoring of anti‐*toxoplasma* antibodies in this population due to the risk of central nervous system damage and many subsequent complications to correct the infection to diagnose and treat.^8^ Although previous studies have discussed the association between IL‐17 and IL‐6 polymorphisms and ocular and congenital toxoplasmosis, there is no study evaluating these polymorphisms in HIV‐positive patients with toxoplasmosis. Therefore, the aim of this study was to analyze and describe the possible effect of polymorphisms localized in the IL‐17A, IL‐17F, and IL‐6 genes in HIV positive patients who infected with *T. gondii* parasite

## 2 MATERIALS AND METHODS

### 2.1 Sampling and data collection

This cross‐sectional study was conducted at the Referral Behavioral Counseling and Modification Center in Shiraz, Iran.

Three hundred thirty‐four patients with HIV positive (which has been confirmed by serological and molecular diagnostic tests) and active cases (patients who have files and regularly and periodically refer to the center for care) were included in the study regardless of gender.

HIV positive patient based on laboratory tests (Toxoplasma IgG ELISA Kit; Pishtazteb www.pishtazteb.com) were divided into two groups: case (with toxoplasma infection) and control (without toxoplasma infection).

*Eligibility criteria*: Any person over the age of 13 years old, who has been confirmed to be HIV positive by laboratory testing and who has been tested for *Toxoplasma* for at least the past 6 months, and who is willing to participate in the study. Individuals who had not been tested for *Toxoplasma* in the last 6 months, or the patients who were in acquired immunodeficiency syndrome stage or for some reason the test results were not recorded on file, or individuals who did not wish to continue the collaboration, were excluded from the study.

The objectives and importance of the study were explained to the participants, and for under 18 years old patients, the consent forms were obtained by their parents.

This study was supported by the Research Vice‐Chancellor of Shiraz University of Medical Sciences with code 22660 and approved by Shiraz University of Medical Sciences with ethical code: IR. SUMS. REC.1400.842.

#### 2.1.1 Sampling

Five milliliters blood samples were taken from all participants. Buffy coats and serum were isolated from blood samples and stored at −70°C until used for DNA extraction, polymerase chain reaction, and restriction endonuclease digestion and cytometric bead array (CBA).

### 2.2 Molecular genotyping method

To analyze genetic polymorphisms in IL‐17A (rs2275913) and IL‐17F (rs763780), and IL‐6 (rs1800795), genomic DNA were extracted from the blood samples.^21^ Four milliliters of whole blood was collected into a tube containing EDTA (Merk) as an anticoagulant. DNA using the salting out method was extracted according to the protocol.^23^ Quantification and assessment of DNA purity was performed using a spectrophotometer (Nanodrop; Thermo Fisher Scientific).

Restriction fragment length polymorphism–polymerase chain reaction (PCR) methods and enzyme digestion ([EcoNI, 10 U/µL, Reference: ER1301] and [NlaIII, 5 U/µL, Reference: ER1831]; Thermo Fisher Scientific) were used to determine the polymorphism of the desired gene regions. 12.5 µL of master mix (Ampliqon2x mastermix red, Denmark,www.ampliqon.com) and 3 µL of DNA, 7.5 µL of deionized water, and 1 µL of each primer were added to each microtube, and PCR was performed. We used primers that designed in previous studies.^24^, ^25^, ^26^

The sequence of primers used for each gene region is as follows.

*IL‐17A*: reverse 5′‐CTCCATAGTCAGAACCCAGC‐3′, forward 5′‐TTGACCCATAGCATAGCAGC‐3′; *IL‐17F*: reverse 5′‐TGGGAATGCAAACAAAC‐3′, forward 5′‐GTGTAGGAACTTGGGCTGCTGCATCAAT‐3′; IL‐6: reverse 5′‐AGTTCATAGCTGGGCTCCTG‐3′, forward 5′‐ CTGCGATGGAGTCAGAGGA‐3′.

After initial testing of the primers, the PCR product was sequenced and approved for quality control. Finally, 7 µL of the PCR product were mixed with 3 µL of the loading solution (Thermo Scientific™ DNA Loading Dye and SDS Solution 6X) and poured into the wells of the 1% agarose gel, followed by electrophoresis at a voltage of 80 V for about 45 min. The intended band of each gene region was checked and confirmed based on the designed primer. Enzymatic digestion was performed on the samples in which the desired band of each gene region that it was selected based on the method of the relevant ANRIMS from the site NEB cutter. Analysis of the digestion products by 2% agarose gel electrophoresis was performed.

#### 2.2.1 CBA for measuring cytokine levels

Serum samples of patients were used to determine the serum level of cytokines. The serum level of IL‐6, IL‐17A, and IL‐17F was measured using a panel designed on the BIOLEGEND website and a flow cytometry device based on the panel procedure.^27^

### 2.3 Statistical methods

Frequency, mean ± SD was used to describe quantitative data. The *χ* ^2^ test was performed to test the relationship between sensitivity to *T. gondii* infection with each genotype and allelic frequencies. The odds ratio was calculated with a 95% confidence interval. The differences in allele and genotype frequencies between patients and control were evaluated with Fisher's exact test. For comparing the difference between observed and expected allelic frequency, the Hardy–Weinberg test was used. The linear regression model was used to analyze the association of IL'SNPs in HIV patients with and without toxoplasmosis. Analysis of haplotypes and linkage test was done using SHEsisPlus software available at http://shesisplus.bio-x.cn/SHEsis.html. A value of *p* < 0.05 was considered statistically significant Table 1.

**Table 1:** RFLP–enzymes, PCR product, and restricted enzyme product size.

Polymorphisms	PCR product size (bp)	Restricted enzyme	Product size (bp)
rs2275913	425	EcoN I	108, 317, 425
rs763780	470	Nla III	52, 130, 288, 418
rs1800795	564	Nla III	29, 111, 122, 129, 173

Abbreviations: PCR, polymerase chain reaction; RFLP, restriction fragment length polymorphism.

## 3 RESULTS

One hundred fifty‐seven women (47%) and 177 men (53%) participated in this study. The mean age of the participants was 44.52 ± 9.62 years. Most of the study participants had middle school education (123, 36.8%) and elementary school education (95, 28.4%), with other levels of education (illiterate,* high school and higher education) ranked next. In terms of marital status, most of the study participants were married, with the next ranks being single, divorced, widowed, and intermittently married. The type of employment of studied people by profession is given in Table 2.

**Table 2:** Number of samples by occupation.

Job categories	Frequency	Percent	Valid percent	Cumulative percent
Jobs outside the home, including seasonal workers, drivers, travel jobs	1	0.3	0.3	0.3
Workers who work in at risk environment, health workers, prison staff, military service members	4	1.2	1.2	1.5
Other government and nongovernment jobs	139	41.6	41.9	43.4
Unemployed	184	55.1	55.4	98.8
Students and college students	4	1.2	1.2	100.0
Total	332	99.4	100.0	
Missing	2	0.6		
Total	334	100.0		

The majority of the studied case were infected with HIV through sexual intercourse (147, 44%) or needle sharing (128, 38.3%). Of all examined subjects, 95 persons (28.4%) had positive result for toxoplasmosis that of them (41.7%) were females, and 55 (57.8%) were males, and this difference was not statistically significant. The mean age in *Toxoplasma*‐negative patients was 43.34 ± 9.78 years, and in *Toxoplasma*‐positive patients was 47.45 ± 8.57 years, and this difference was statistically significant (*p* = 0.001). The results of the statistical tests indicated that *Toxoplasma* infection was significantly related to literacy level (*p* = 0.009). Other variables, such as tranmission HIV, marital status, job, are unrelated to getting *Toxoplasmosis*.

Regression analysis of IL‐17 and IL‐6 polymorphisms in different dominant, recessive, and over dominant states showed no significant association with toxoplasmosis infection rate. The separate results are shown in Table 3.

**Table 3:** Regression analysis of IL‐17A, IL‐17F, and IL‐6 SNP genotypes in HIV patients with and without toxoplasmosis.

SNP	Genetic model	Genotype	Cases *N* (%)	Control *N* (%)	OR (95% CI)	*p* Value
IL‐17A (rs2275913)	Dominant	GG AG + AA	5 (5.2%)	19 (8%)	1.579 (0.572–4.357)	0.378
			91 (94.8%)	219 (92%)		
	Recessive	AA GG + AG	75 (78.1%)	188 (79%)	1.053 (0.592–1.872)	0.861
			21 (21.9%)	50 (21%)		
	Over dominant	AG GG + AA	26 (27.1%)	69 (29%)	1.099 (0.647–1.868)	0.727
			70 (72.9%)	169 (71%)		
	Codominant	GG	5 (5.2%)	19 (8%)	–	0.677
		AG	70 (72.9%)	169 (71%)	1.574 (0.565–4.381)	0.385
		AA	21 (21.9%)	50 (21%)	1.596 (0.526–4.839)	0.409
IL‐17F (rs76378)	Dominant	AA GA + GG	48 (50%)	106 (47.7%)	1.017 (0.633–1.634)	0.945
			48 (50%)	116 (52.3%)		
	Recessive	GG GA + AA	94 (97.2%)	6 (2.7%)	1.667 (0.274– 10.134)	0.579
			2 (2.1%)	216 (97.3%)		
	Over dominant	GA AA + GG	50 (52.1%)	110 (49.5%)	0.984 (0.612–1.581)	0.947
			46 (47.9%)	112 (50.5%)	1.515	
	Codominant	AA GA GG	48 (50%)	106 (47.7%)	–	0.857
			46 (47.9%)	110 (49.5%)	1.000 (0.620–1.614)	1.000
			2 (2.1%)	6 (2.7%)	1.667 (0.270–10.289)	0.582
IL‐6 (rs1800795)	Dominant	GG GC + CC	78 (81.3%)	182 (82%)	0.733 (0.405–1.326)	0.304
			18 (18.8%)	40 (18%)		
	Recessive	CC GC + GG	81 (84.4%)	34 (15.3%)	0.631 (0.336–1.183)	0.151
			150 (15.6%)	188 (84.7%)		
	Over dominant	GC GG + CC	93 (96.9%)	6 (2.7%)	2.527 (0.501–12.746)	0.262
			3 (3.1%)	216 (97.3%)		
	Codominant	GG GC CC	78 (81.3%)	182 (82%)	–	0.212
			3 (3.1%)	6 (2.7%)	2.321 (0.485–11.751)	0.309
			15 (15.6%)	34 (15.3%)	0.645 (0.343–1.212)	0.172

Abbreviations: CI, confidence interval; HIV, human immunodeficiency virus; IL‐17A, interleukin‐17A; OR, odds ratio; SNP, single‐nucleotide polymorphism.

IL‐17 and IL‐6 allele frequencies are shown in Table 4. These results also showed no significant difference in the different allelic polymorphisms.

**Table 4:** Allele frequencies of IL‐17A, IL‐17F, and IL‐6 polymorphisms in HIV patient with and without toxoplasmosis.

SNP		Control (239)		Case (95)		*p* Value
	Allele	*n*	%	*n*	%	
IL‐17A(G/A)	G	104	43.5	39	41.0	0.73
rs 2275913	A	135	56.5	56	59.0	
IL‐17F(A/G)	A	178	74.5	70	73.7	0.93
rs 763780	G	61	25.5	25	26.3	
IL‐6(G/C)	G	183	76.6	78	82.1	0.17
rs 1800795	C	56	23.4	17	17.9	

Due to the fact that, based on the linkage test, two polymorphisms of the examined IL‐17 polymorphisms were completely correlated with each other (*R* = 1, *D* = 1), a haplotype analysis was carried out for them. There was no difference between haplotypes and toxoplasmosis rate. The results are shown in Table 5.

**Table 5:** Estimated haplotype frequencies and odds ratio (OR) between SNPs—rs2275913 and rs763780—located in IL‐17 in HIV patients with and without toxoplasmosis.

Haplotype	Case (*N*)	Control (*N*)	*χ* ^2^	Fisher's *p*	Pearson's *p*	OR [95% CI]
A‐A	69 (0.363)	170 (0.355)	0.033	0.858	0.855	1.033 [0.728–1.466]
G‐G	7 (0.036)	22 (0.046)	0.276	0.679	0.599	0.792 [0.332–1.888]
A‐G	69 (0.363)	170 (0.355)	0.033	0.858	0.855	1.033 [0.728–1.466]
G‐A	45 (0.236)	116 (0.242)	0.025	0.920	0.873	0.968 [0.652–1.436]

Abbreviations: CI, confidence interval; HIV, human immunodeficiency virus; IL‐17, interleukin‐17; SNP, single‐nucleotide polymorphism.

**Figure 1:**
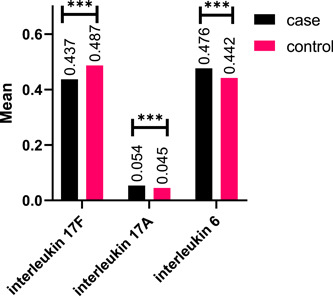
Mean of interleukins 17A, 17F, and interleukin 6 serum levels in case and control groups. ***, statistically significant in 0.05 level.

Measurement of the serum levels of IL‐17A, IL‐17F, and IL‐6 showed that the average serum concentration of these cytokines in non *Toxoplasma* infected cases and those infected with *Toxoplasma* has a significant difference. Thus, the average concentration of IL‐6 and IL‐17A in the case group (0.476 ± 0.012 and 0.054 ± 0.006) was significantly higher than in the control group (0.442 ± 0.048 and 0.045 ± 0.018). However, in the case of IL‐17F, the plasma concentration of the control group (0.487 ± 0.281) was higher than that of the case group (0.437 ± 0.042) (Figure 1).

The results of genotype analysis of IL plasma concentration by genetic models in case and control groups are presented in Table 6. The results showed that with the exception of IL‐17A and IL‐F, which was not significant in the recessive state, the difference in the rest of the genetic state in the case and control groups was significant.

**Table 6:** Genotype analysis of IL‐17A, IL‐17F, and IL‐6 serum level in HIV patient with and without toxoplasmosis.

Cytokine serum level	Genotype	Cases mean ± SD	Control mean ± SD	*P* value
IL‐17A	GG	0.050 ± 0.020	0.040 ± 0.004	0.006*
	AA	0.050 ± 0.010	0.040 ± 0.004	0.001*
	AG	0.050 ± 0.004	0.040 ± 0.020	0.001*
IL‐17F	AA	0.440 ± 0.060	0.480 ± 0.030	0.001*
	GA	0.430 ± 0.010	0.480 ± 0.020	0.001*
	GG	0.430 ± 0.000	0.490 ± 0.000	0.046*
IL‐6	GG	0.470 ± 0.010	0.440 ± 0.050	0.001*
	CC	0.480 ± 0.000	0.440 ± 0.000	0.001*
	GC	0.480 ± 0.000	0.440 ± 0.000	0.025*

Abbreviations: HIV, human immunodeficiency virus; IL‐17A, interleukin‐17A.

* Statically significant.

## 4 DISCUSSION

Toxoplasmoa infection is one of the major problems that disrupts the control process of HIV patients. Many immunological factors, including cytokines, play a protective role in contracting infectious diseases. Because these ILs exhibit multiple polymorphisms, and each of them may play a role in regulating the functions of ILs, in this study we showed a significant differences of IL‐6 and IL‐17A serum level, between the case and control groups in various genetic models.

In total, 95 patients (28.4%) tested positive for toxoplasmosis, and the findings were consistent with previous research, particularly in the same geographic area.^5^, ^22^, ^28^, ^29^

However, when compared to a specific study, our results were less encouraging.^30^, ^31^, ^32^

This variation can be linked to variances in geographical areas, weather conditions, and animal contact, as previously indicated in earlier studies. Furthermore, risk factors such as occupation, gender, residence, and level of education can have an indirect effect on the pattern of infection spread. In terms of the demographic index, there was a significant correlation between age and *Toxoplasma* infection.

Those infected with the *Toxoplasma* parasite are generally older than those who are not. The current study's findings are consistent with Rezanezhad and colleagues,^28^, ^31^, ^33^ it was observed that there is a significant link with *Toxoplasma also* the rate of infection increases with age. This correlation in the study of Nissapatorn, Ahmadpour, and Xavier was not significant.^8^, ^29^, ^30^ This disagreement in the results could be described to differences in group division, sample quantity, and *toxoplasma* detection laboratory method.

Furthermore, the current study found a link between education and getting infected with this parasite. The persons with a lower level of education being more likely to be infected with toxoplasmosis. A substantial difference was identified between education and the likelihood of acquiring toxicosis in the study of Xavier et al.^8^ which was similar to our results. This association was insignificant in studies,^15^, ^28^, ^29^ and this discrepancy may be attributed to differences in group classification.

In this study, there was no discernible difference between the case and control groups for indicators of occupation, gender, marital status, or transfer method. The findings are in line with Rezanezhad and colleagues studies, and neither one has found any evidence of a gender or marital status effect that would be considered statistically significant.^3^, ^15^, ^28^

In Ahmadpour et al.^29^ and Wujcicka's et al.^15^ studies, there is no correlation between person's job and their risk of contracting *toxoplasma*. Also, in the study of Rahimi et al.^3^ and Bokharaei‐Salim,^31^ like our study, no significant relationship between the mode of transmission and toxoplasmosis was reported.

The risk of *toxoplasma* infection in the current study did not correlate with IL‐17 polymorphism in the two gene regions rs2275913 and rs763780.

According to the finding of this research by de Araujo Andrade et al. Allelic studies revealed that the G allele in the IL‐17F polymorphism has a more protective role than the C allele, in pregnant women,, there was no significant corrolation between the polymorphism of IL‐17F and the likelihood of contracting Toxoplasma.^1^

Regarding the association between IL‐17 polymorphism and the possibility of contracting toxoplasma in the population of HIV‐positive patients, no comparable research has been done.

The polymorphism of IL‐6 and the risk of contracting toxoplasma were also not significantly correlated in this study. Studies have been done on this gene region in toxoplasma that affect the ocular and neonatal, as well as in various target populations like pregnant women and the general public.A significant correlation between the GG genotype and the risk of congenital toxoplasmosis has been demonstrated in Wujcicka's study.^34^ The C allele, however, was significantly associated with adult ocular toxoplasmosis and congenital infection in a study done by the same author in 2018.^15^

IL‐6 polymorphism and ocular toxoplasmosis are significantly correlated, according to Cordeiro's study. The relationship between IL‐6 polymorphism and the risk of toxoplasmosis in HIV‐positive patients has notfound, however, been the subject of similar studies.^17^

Given that IL‐17A and IL‐17F were linked, and the linkage test revealed that these two gene loci are linked together (*D*′ = 1 and *r* = 1), the linkage test for these two ILs was carried out, and it once more revealed a significant relationship.

There was no association between the frequency of alleles and the risk of *toxoplasma* infection in IL‐17 haplotype analysis. There hasn't been any research of this kind in the area.

The effects of linkage analysis on IL‐17A and IL‐17F in HIV patients have not been investigated in a study of a similar nature.

The current findings demonstrated a significant difference between the group of non Toxoplasma infected individuals and those who were infected with Toxoplasma in terms of the average concentration of the serum level of these cytokines. In such a way that the case group's average IL‐6 and IL‐17A concentration was significantly higher than that of the control group. However, in the case of IL‐17F, the control group's plasma concentration was greater than that of the case group.

The research by Satti et al. according to the findings, IL‐17 levels in the blood were typically significantly higher in Toxo patients than in healthy controls. These outcomes match those of the current study.^35^ Additionally, Evangelista et al.'s research revealed that toxo positive individuals had plasma concentrations of IL‐17A that were significantly higher than those of healthy individuals.^36^

The findings of the study,^37^ however, did not match those of the current study because, in this study, toxoplasma patients had significantly lower serum levels of IL‐6 than the control group. This difference might be the result of sampling within a particular group.

Our findings demonstrated that in various genetic models (recessive, etc.), there was a significant difference between the case and control groups' serum levels of IL‐6 and IL‐17A but not IL‐17F.

Wujcicka et al.'s findings support this. Minor allele c of IL‐6, compared to healthy individuals, significantly correlates with elevated cytokine levels in Toxo‐infected individuals.^15^

In addition to the findings discussed above, the linkage analysis performed on the data suggests that the genetic polymorphisms of the two regions, IL‐17F and IL‐17A, which are located on the same locus, are closely related to one another and transmitted together and separate investigations of each of these polymorphisms may yield contradictory findings. Therefore, as shown in Table 5, the outcomes of genetic studies relating to IL‐17F and IL‐17A frequently have polar opposite outcomes. For instance, it appears that these cytocines as proinflammatory factors should increase during infection; IL‐6 and IL‐17A follow this rule, but IL‐F exhibits decreasing results in the study of the plasma concentration of secreted cytokines in toxoplasma patients.

In terms of structural research, we also know that IL‐17F is found in the up stream of IL‐17A according to the genetic sequence, and it appears that in addition to the regulatory function of IL‐17A on IL‐17F, so polymorphism 17A investigation alone is sufficient.

In Iran, this is the first investigation into the role of IL‐6 and IL‐17 polymorphism in toxoplasma infection in HIV‐positive patients.

## AUTHOR CONTRIBUTIONS

**Maryam Nejabat**: conceptualization (equal); investigation (equal); writing—original draft (equal); writing—review and editing (equal). **Mohammad Motamedifar**: Conceptualization (equal); supervision (equal); project design. **Zohre Foroozanfar**: Formal analysis (equal); methodology (equal); project administration (equal). **Mohammadreza Heydari**: Data curation (equal); supervision (equal); writing—original draft (equal); writing—review and editing (equal). **Saeid Amiri Zadeh Fard**: Writing—review and editing (equal); methodology (equal). **Ava Hashempour**: Writing—review and editing (equal); project administration (equal). **Nazani Nazari**: writing—review and editing (equal); methodology (equal). **Esmaeil Rezaei**: Project administration (equal); lab analysis (equal). **Zahra Heydari**: Project administration (equal); lab analysis (equal); editing (equal). All authors reviewed and approved the final version submitted for publication Data.

## CONFLICT OF INTERESTS STATEMENT

The authors declare no conflict of interest.

## Data Availability

n/a.

## Footnotes

* Inability to read and write.
